# Biotransformation technology and high-value application of rapeseed meal: a review

**DOI:** 10.1186/s40643-022-00586-4

**Published:** 2022-09-24

**Authors:** Zhengfeng Yang, Zunxi Huang, Lijuan Cao

**Affiliations:** 1 Engineering Research Center of Sustainable Development and Utilization of Biomass Energy, Ministry of Education, Yunnan Normal University, Kunming, 650500 People’s Republic of China; 2 School of Energy and Environmental Science, Yunnan Normal University, Kunming, 650500 People’s Republic of China; 3 Key Laboratory of Yunnan for Biomass Energy and Biotechnology of Environment, Yunnan Normal University, Kunming, 650500 People’s Republic of China; 4 College of Life Sciences, Yunnan Normal University, Yunnan Normal University, No. 768 Juxian Street, Chenggong, Kunming, Yunnan 650500 People’s Republic of China

**Keywords:** Rapeseed meal, Antinutritional factors, Biotechnology, High-value biotransformation

## Abstract

Rapeseed meal (RSM) is an agro-industrial residue of increased functional biological value that contains high-quality proteins for animal feed. Due to the presence of antinutritional factors and immature development technology, RSM is currently used as a limited feed additive and in other relatively low-value applications. With increasing emphasis on green and sustainable industrial development and the added value of agro-industrial residues, considerable attention has been directed to the removal of antinutritional factors from RSM using high-efficiency, environment-friendly, and cost-effective biotechnology. Similarly, the high-value biotransformations of RSM have been the focus of research programmes to improve utilization rate. In this review, we introduce the sources, the nutrient and antinutrient content of RSM, and emphasize improvements on RSM feed quality using biological methods and its biotransformation applications.

## Background

Besides soybean, oilseed rape is an important oil crop worldwide, and rapeseed meal (RSM) is the byproduct of rapeseed oil production (Landero et al. 2012). RSM is characterized by high yield, with rich protein content (35–44%), good amino acid balance, and low price. Recent increases in the prices of the main protein sources for animal feed combined with a major shortage of feed protein resources have placed the global fodder industry under great pressure. Therefore, the livestock farming industry is faced with an urgent need to obtain other excellent feed protein sources to fill the ever-expanding demand gap. This has become an urgent issue that requires resolution at the global level. The antinutritional factors contained in nondetoxified RSM not only affect feed digestibility and the utilization of nutrients, but also cause adverse effects on animal health, which greatly limit the amount of RSM as feed and result in a huge waste of RSM resources (Xie et al. 2012). Therefore, the development of RSM with a low antinutritional content and high nutritional value at a low cost has become the main aspect of research on feeding RSM (Ghodsvali et al. 2005; Slominski et al. 2012).

**Fig. 1:**
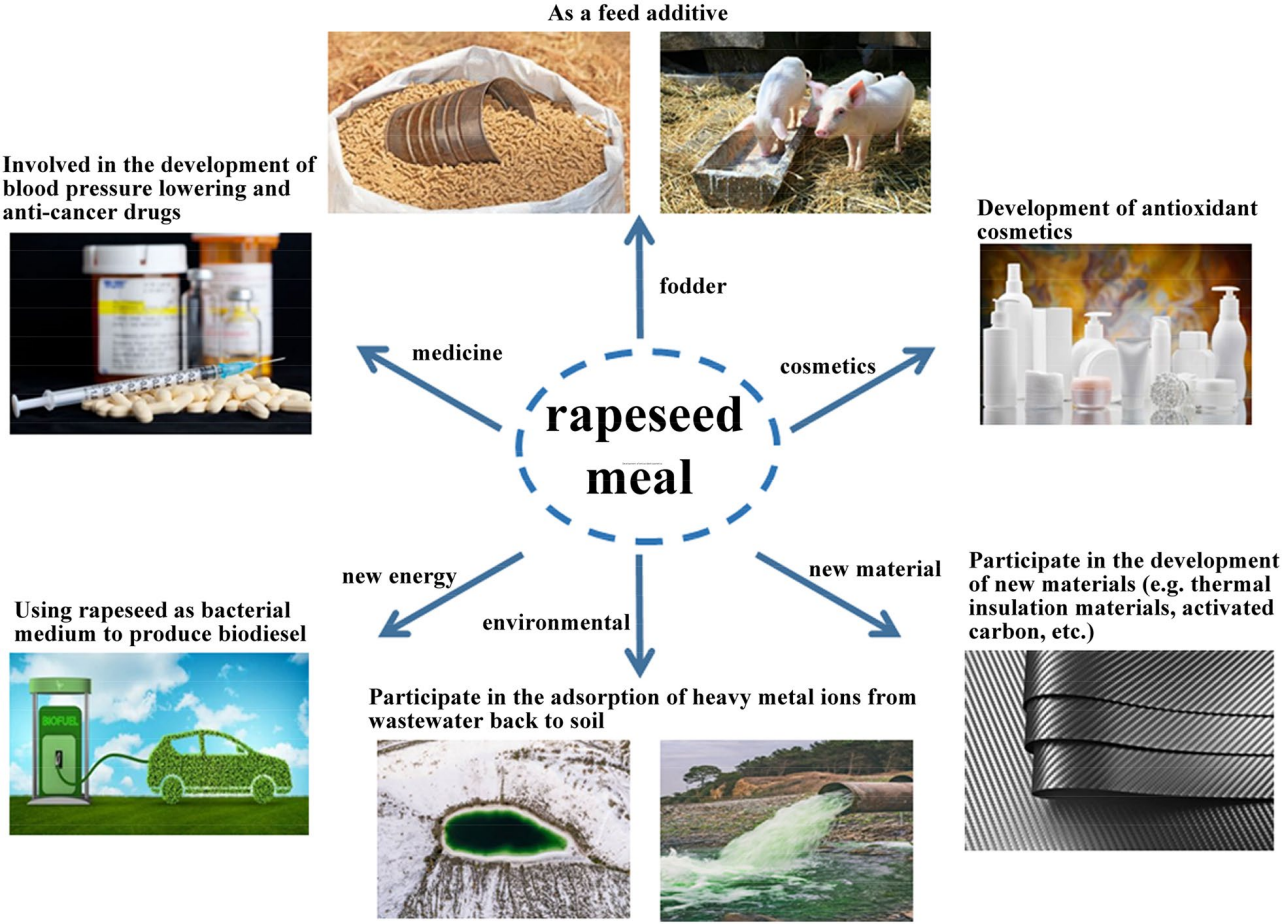
Application of rapeseed meal and potential development of high value (picture materials related to the application of rapeseed meal (such as cosmetics) were purchased from Paixin (https://www.paixin.com/)

One of the strategies for sustainable waste management is recycling or waste treatment to create value-added products. For example, the biological transformation and utilization of jujube processing waste, tea waste, and lobster processing byproducts have been reported (Oladzad et al. 2021; Guo et al. 2021; Nguyen et al. 2017). Currently, RSM treatment mainly includes two strategies (Fig. 1): the use of safe microorganisms or enzymes, combined with environment-friendly and cost-effective physical and chemical methods, to remove antinutritional factors and improve the utilization rate of RSM in feed; and the compounds in RSM can be biotransformed to obtain RSM protein, active peptides, antioxidant, and other bioactive substances by Enzymolysis or fermentation, which will expand the potential value of RSM in fermentation, food, medical, energy development, new materials, cosmetics, and other applications.

Given the high commercial and application value of RSM in the feed industry, we have witnessed a rise in studies that focus on RSM. However, at present, detailed reviews on improving the quality of feeding RSM using biological methods and high-value biotransformation technology and their applications are lacking. Hence, this paper reviews aspects of the nutritional and antinutritional content in RSM as well as research progress in the biotransformation, biotechnology, and high-value uses of RSM to provide a theoretical basis and technical reference for the improvements and development of future applications of RSM.

## Sources, nutrition, antinutritional factors, and toxicity mechanism of RSM

### Sources

Rapeseed is the most commercially viable genus of Brassicaceae and one of the major oil crops worldwide. Oilseed rape comprises four species: *Brassicaceae napus* L., *B. juncea* L. *Brassica campestris* L. and Ethiopia rapeseed; of these, *B. napus* is the most common species due to its strong adaptability and planting range, resulting in a wide distribution across the six continents. RSM is a byproduct of the rapeseed oil industry, accounting for 90% of rapeseed byproducts, and is manufactured using various processes, such as pressing and leaching. In Germany, rapeseed oil byproducts rank second in vegetable protein content after soy products (Kracht et al. 2004). In 2017, global output reached 40.51 million tonnes, making it the second largest output of protein cake (USDA 2017). Changes in RSM yield are mainly determined by changes in rapeseed yield. Generally, ~ 60% of RSM is obtained by processing rapeseed via modern methods, with different processing techniques yielding different types and qualities of RSM. Depending on the processing temperature, RSM can be divided into high-temperature meal, intended for traditional baking, pre-pressing, and leaching, and low-temperature meal destined for low-temperature cold pressing (Kracht et al. 2004). Compared with high-temperature RSM, low-temperature RSM appears to be superior for animal feeding. Prolonged high-temperature exposure of rapeseed during oil production can greatly reduce the feeding value of RSM by partly degrading protein content and increasing the level of indigestible cellulose. Watts et al. (2020) obtained RSM with different nutritional values using supercritical carbon dioxide extraction (ScCO2) and cold-pressed hexane extraction (CpHe). Compared with CpHe, ScCO2-produced RSM showed higher metabolizable energy, better protein digestibility, and energy metabolic rate during broiler feeding due to less heat damage (Mosenthin et al. 2016). Currently, classification distinguishes between double-low and common RSM, depending on the content of erucic acid and glucosinolate. Canola meal is a typical example of double-low RSM, which contains < 2% erucic acid and < 30 µmol/g glucosinolate. Common RSM is richer in antinutritional factors and has poor palatability; however, its protein solubility is higher, and its price is maintained lower.

### Nutrient composition

RSM contains ~ 3.5% crude fat, 35% crude protein, 6% crude ash, and 12% crude fibre. In addition, it contains ~ 4% phytic acid, 15 mmoL/g glucosinolates (GLS), polyphenols, and several essential amino acids, such as arginine, methionine, and lysine (Gatlin III et al. 2007; Yu et al. 2015). Its content of methionine and cysteine is higher than that of soybean (Khajali et al. 2015), although lysine content is slightly lower. RSM is rich in mineral elements, such as calcium, phosphorus, magnesium, and selenium; importantly, the content of selenium is the highest among vegetable proteins (Kissil et al. 2000). Selenium deficiency is a common global issue, which raises the importance of RSM in potential commercial applications. RSM is also a good source of vitamins, particularly the vitamin B complex (niacin, folic acid, B1, and B2), and the essential nutrient choline (Seneviratne et al. 2010).

The amino acid composition of rapeseed protein is reasonable and within the levels recommended by the WHO/FAO. Its nutritional value is equal to or better than that of animal protein; therefore, it is considered an excellent plant protein (Gorissen et al. 2018). Jones (1979) reported that the protein efficiency ratio of rapeseed protein was better than that of casein, and Ohlson and Anjou (1979) showed that the net utilization ratio of rapeseed protein was 87–90%. Ingestion of RSM protein results in a decreased insulin response after meals and stronger satiety than soybean protein (Volk et al. 2020). Furthermore, hydrolysis of the rapeseed protein produces a mixture of low-molecular-weight peptides and various amino acid chain lengths, including active peptides with important physiological functions that could be further explored by the pharmaceutical and food industries. Compared with those of other proteins, rapeseed peptides exhibit good acid solubility, low viscosity, gel formation resistance, and solubility (90% higher than rapeseed protein solubility) in addition to higher absorption and utilization rates (Pinterits and Arntfield 2008; Chabanon 2007).

### Antinutritional factors and toxicity mechanism

**Fig. 2:**
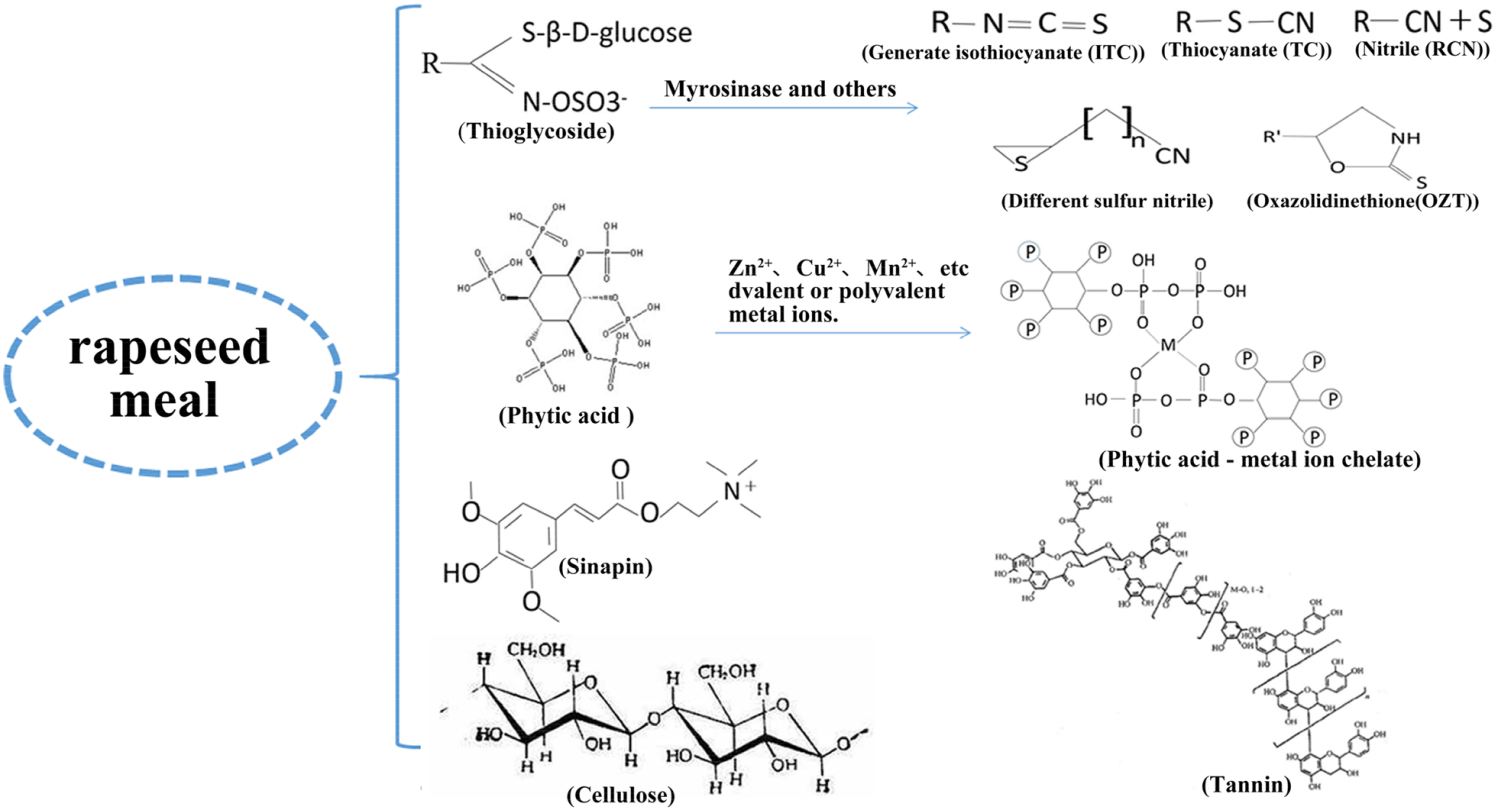
Antinutritional factors and toxicity mechanism of rapeseed meal

RSM is considered a resource of great potential industrial value due to its vast reserves and high nutritional content. However, at present, its development and application are largely limited by the elevated content of antinutritional factors (Fig. 2). Glucosinolates, phytic acid, tannin, sinapine (SE), cellulose, and lignin are the main antinutritional factors of RSM, can reduce nutrient absorption of RSM as animal feed.

### GLS

GLS comprise a group of secondary metabolites that are widely accumulated in *Brassica* plants, especially in the reproductive organs (Grubb and Abel 2006). GLS themselves are nontoxic; they play an important role in defence mechanisms of plants against diseases and have been used in antioxidant applications for humans. In the animal gastrointestinal tract, exogenous myrosinase catalyses the hydrolysis of GLS into glucose and an unstable intermediate, β-aglycone, which spontaneously transforms to isothiocyanate (ITC), oxazolidine thione (OZT), thiocyanate (TC), and nitriles (RCN), depending on the environment (Sonderby et al. 2010; Roberta et al. 2003); of these compounds, nitrile is the most toxic, ~ 5–10 times more toxic than OZT. TC and ITC are similar in shape and size to iodine; when present in excess levels in the blood, they compete with iodine for uptake in the thyroid gland and inhibit iodine transport into the thyroid follicular cells; while OZT too has similar characteristics. In animals, these compounds may cause diarrhoea, haemorrhagic gastroenteritis, and goitre, and, in extreme cases, liver haemorrhage and liver necrosis, thereby endangering health and hindering growth (Schone et al. 1997; Tanii et al. 2004). Heat treatment can effectively remove GLS from RSM, but the method is limited by its high specificity for heat-sensitive factors. In addition, heat treatment can trigger a Maillard reaction between lysine and starch and reduce the nutritional properties of other substances.

### Phytic acid

Phytic acid (cyclohexanol-β-phosphate), a strong chelating agent, is an important antinutritional factor in RSM; its content is generally 2–5%. Phytic acid firmly chelates positively charged divalent or polyvalent metal ions, such as zinc, calcium, copper, magnesium, manganese, and iron, to form insoluble phytates, thereby reducing the biological efficiency of some essential mineral elements. In addition, it binds to proteins to form insoluble complexes, greatly reducing the biological potency and digestibility of proteins. In animals, phytic acid affects a series of digestive enzymes, such as proteases, amylases, and lipases, and thereby impacts digestion and nutrient absorption (Ravindran et al. 2000). Due to its relatively stable chemical properties, phytic acid cannot be degraded by physical treatments, such as heating, and there is no corresponding animal enzyme system to digest and degrade it. At present, phytase is being widely used to break down phytic acid.

### Phenolic compounds (including tannins and SE)

Tannin is mainly distributed in the rapeseed hull. Its average content amounts to ~ 3.65%, and is responsible for the bitter taste and poor palatability of rapeseeds (Butler et al. 1984). Tannin binds to enzymes in the digestive tract to form inactive compounds, and to dietary proteins, forming insoluble compounds, thereby affecting the digestion of proteins and other nutrients (Reddy et al. 1985). In addition, it interferes with the bioavailability of mineral elements by facilitating the precipitation of various metals, such as ionized calcium, iron, and zinc, thus reducing their utilization rate, which in turn affects animal growth and feed conversion. Tannin is easily oxidized and polymerized under neutral or alkaline conditions, resulting in the black colour and bad odour of hulls (Amarowicz et al. 2000). Some studies have identified microbes that can degrade tannins. For example, Nelson et al. (1997) reported that diplococci bacteria degraded tannins under anaerobic conditions, at a rate of 30 g/L of tannins in ~ 3–4 h. Osawa et al. (2000) isolated tannin-degrading bacteria from human faeces. Mcsweeney et al. (2001) found that *Clostridium botulinum* could use tannin as the sole carbon source.

Obied et al. (2013) analysed the composition of phenolic antinutritional substances in Canadian RSM and found that the SE content was the highest, accounting for ~ 80% of the total phenolic content in rapeseed. Such large amounts of SE are responsible for the bitterness of cake meal. Landero et al. (2012) demonstrated that the bitterness of cake meal affected food intake by pigs, thus directly influencing average daily feed intake and weight gain. Degradation of SE involves cleavage of its ester structure through the enzymatic action of polyphenol oxidase, tannase, and tyrosinase. Hu et al. (2004) showed that laccase significantly reduced SE content in solid-state fermentation. It has been pointed out that the polyphenol oxidase produced by white-rot fungi fermentation can decompose > 98% of SE and sinapic acid in aqueous solutions (Lacki and Duvnjak 1998).

### Crude fibre

Crude fibre in RSM is mainly found in the shell, where its content ranges from 9–20%. The presence of crude fibre, which cannot be digested and absorbed by animals, may prevent contact between intracellular nutrients and enzymes in the gastrointestinal tracts of animals and reduce the digestibility of feed. This is considered the primary reason for the low digestibility and metabolic energy of RSM (Bell 1993). Removal of crude fibre is commonly achieved through treatment with enzymes and microorganisms.

## Comparison of methods to improve RSM quality

In recent years, the value of RSM as a feed additive has drawn considerable attention. Elimination of antinutritional factors and improvement of the nutritional value of RSM have become primary research directions. At present, rapeseed detoxification is mainly achieved using physical, chemical, genetic (breeding-based), and biological methods. Different detoxification methods are associated with specific technological characteristics and effects (Table 1).

**Table 1:** Comprehensive evaluation of different processes and their efficacy in rapeseed detoxification

Modes of action	Technology	Description of methods	Effectiveness evaluation	References
Physical detoxification	Heat	Degradation by high temperature	Not obvious	Jensen et al. (1995)
	Radiation	Inactivate myrosinase by radiation and decompose part of phytic acid and tannin	Not obvious	Maheshwari et al. (1980)
	Extrusion and expansion	The raw materials are expanded by steam, electricity, or sudden decompression after being heated by extrusion friction	Obvious	Nibedita et al. (2007)
	Hulling	Break and peel	Not obvious, but it improves nutrition	Kracht et al. (2004)
Chemical detoxification	Acid–base treatment	Soak or heat the rapeseed meal with acid–base solution	Obvious, but the feeding nutritional value is reduced	Bhatty and Sosulski (1972) Manashi et al. (2014)
	Salt treatment	Chelation of free cations in salt with hydrolysate of antinutritional factors	Obvious, but the nutrition and palatability are reduced	Das and Singhal (2005)
Biological detoxification	Genetic breeding	Using different techniques to improve rapeseed quality at gene level	Obvious, the nutrition is improved	Hannoufa et al. (2014)
	Enzyme addition	Added directly	Obvious, the nutrition is improved	Xue et al. (2009b)
	Microbial fermentation	Using the complex enzyme system secreted by microorganism itself	Obvious, and the nutritional value is significantly improved	Wang et al. (2012)

Chemical methods include acid or alkali treatments. These can be supplemented with ammonium sulfate reagents and methanol, ethanol, isopropyl alcohol, ethane, and other dehydration solvents for precipitation or extraction separation. Several studies have reported data on protein isolation and removal of antinutritional factors. For example, Berot and Briffaud (1983) treated RSM with 60% (v/v) ethanol or isopropanol, with a resulting increase in the protein concentration of dry matter to 63 g and removal of 97% of polyphenols and 99% of GLS. Chabanon et al. (2007) were successful in removing 75% of GLS from RSM using ethanol extraction. Over 90% of GLS can be separated using the reverse micellar method, but the process is complicated and requires introduction of a surfactant (such as Triton-X100 and Twin-85). Alkali extraction combined with membrane filtration can reduce the content of GLS, phytic acid, and other substances; however, the membrane filtration equipment is expensive and difficult to maintain (Aider and Barbana 2011). Moreover, the functional tertiary structure of proteins is inevitably damaged after treatment with acids or alkali, leading to loss of biological activity and reducing the bioavailability of RSM and its application value as feed and functional food (Baker and Charlton 2020).

Physical methods primarily adopt extrusion, ultrasonication, hulling and other strategies to process RSM. Extrusion can increase the solubility of non-starch polysaccharides and the accessibility of enzymes, but has no obvious effect on the digestion of fibrous polysaccharides and effects of processing on glucosinolates and myrosinase activity were minor (Vries et al. 2014). Hulling treatment can reduce the content of cellulose in RSM, but cannot reduce the content of antinutritional factors such as GLS and sinapine (Kracht et al. 2004). These processes are primarily characterized by low efficiency and single effect, reducing the protein quality of RSM and leading to denaturated and deactivated beneficial substances. Heat treatment is a popular method aimed at removing antinutritional factors. Although > 70% of GLS in RSM can be degraded using this method, heat treatment can also lead to decomposition products similar to those catalysed by myrosinase, affecting feed quality (Campbell and Slominski 1990). Overall, the detoxification range and environmental effects of the physical and chemical methods for the removal of antinutritional factors from RSM are not optimal. In addition, the palatability of rapeseed cake is negatively impacted, which seriously reduces the utilization rate and nutritional characteristics of rapeseed protein (Vig and Walia 2001). Therefore, physical and chemical techniques for the mass industrial production of high-quality RSM are still lacking. Kumar and Sharma (2017) indicate that the choice of pretreatment depends completely on the application and types of waste. For example, compared with the traditional single-pretreatment technology, the processing of lignin involves a combination of two or more pretreatment processes. Thus, an integrated strategy is not only beneficial in minimizing the production of harmful inhibitors but also in reducing the number of processing steps. Biochemical transformation begins with a low degree of thermochemical pretreatment to partially destroy cell walls and expose cellulose and hemicellulose components to improve enzyme accessibility (Kumar and Sharma 2017).

Currently, biological detoxification appears to be the most researched approach for improving RSM quality, locally and internationally. Due to its wide detoxification range and high detoxification rate, biological methods show potential for effectively enhancing the palatability and improving the nutritional value of RSM products. Importantly, biological approaches are considered optimal for improving the quality of RSM and are consistent with green, pollution-free, and sustainable development. Thus, in the following sections, we focus on the various biological detoxification methods, such as enzyme addition and microbial fermentation.

## Improving RSM quality using biological methods

### Enzymolysis

Compared with traditional physicochemical methods, enzyme-assisted methods for the removal of antinutritional factors have attracted considerable attention because of their high efficiency, benign sustainability, and ecological friendliness (Table 2). Enzymatic extraction relies on the characteristics of enzymes and is highly specific. In addition, it shows characteristics of regional selectivity and reaction under mild conditions, while preserving the potency of biological compounds (Nadar et al. 2018). Currently used enzymes comprise alkaline, neutral, and acidic proteases, trypsin, papain, enzymes that mediate the production of flavour compounds, laccase, oxidative polyhydrogenase, and cell wall polysaccharide-degrading enzymes. Alkaline protease is the most commonly used enzyme, with a good hydrolytic capacity; its activity is matched by enzymes enhancing flavour compounds that can improve the degree of hydrolysis and peptide yield of RSM, eliminate the bitterness of RSM, and increase palatability.

**Table 2:** Different biological methods used to improve the quality of rapeseed meal and its effect evaluations

Modes	Enzymes	Microorganisms	Conditions	Evaluations of nutrition	References
Enzyme	Alcalase, flavourzyme		Alcalase: 50 ℃, 1 h, then flavourzyme: 2 h	After digestion by flavourzyme, DH increased to 30%, bitterness decreased by 60%	Xue et al. (2009a, b)
	Alcalase		Ultrasonic, then Alcalase	The protein hydrolysis rate of rapeseed was increased	Jin et al. (2016)
	Cellulase, pectinolytic, xylanolytic		Cellulase and xylanase: 50 ℃, pectinase a: 30 ℃ and pH 5.5, 48 h	The extraction rate of protein increased by 73% and 58%, respectively, which significantly improved the decomposition of polysaccharide	Rommi et al. (2014)
	Cellulase, pectinase		cellulase, two pectinase and alkaline	Increased the degradation of fibre in RSM	Long et al. (2020)
	Hemicellulase, pepsin, papain, trypsin, ficin		40 ℃ for 6 h	Antinutritional factors decreased significantly, improving the solubility of nitrogen	Mahajan and Dua (1998)
	Phytase		24 h solid fermentation at 30℃ under anaerobic conditions	Degrades more than 80% of carbohydrates, 30% of lignin and 45% of total GLS, significantly improve its nutrition	Drażbo et al. (2020)
	Phytase		75 ℃ and pH 12.5	Phytic acids are decreased by about 25%	Rodrigues et al. (2017)
	Xylanase		Direct addition of enzymes	Significantly increased ileum digestibility and total digestibility of nutrients	Fang et al. (2007)
Microorganisms		*Aspergillus niger*	After mixing 80% RSM and 20% wheat bran, *Aspergillus Niger* fermented for 72 h	The small peptide is 2.26 times larger than the unfermented RSM. The decomposition rates of antinutritional substrates such as neutral fibre (NDF), GLS, isothiocyanates, oxazolidinone and phytic acid, were increased by 13.47, 43.07, 55.64, 44.68 and 86.09%	Vig et al. (2001)
		*Aspergillus terreus*, *Lichtheimia* sp. JN3C, *Yeast*	Solid-state fermentation for 96 h	The degradation rate of crude fibre, phytic acid, total GLS and protein was 66.2%, 28.3%, 98% and 27.4%	Wang et al. (2012)
		*Aureobasidium,* *pullulans, A. pullulans,* *Trichoderma reesei,* *Fusarium venenatum,* *Pichia kudriavzevii* *and Mucor* *circinelloides*	Solid fermentation	The content of protein increased greatly, and the content of GLS decreased significantly	Croat et al. (2016)
		*Bacillus subtilis*	Inoculation amount is 5% (v/v), fermentation temperature is 28℃, pH 7.0, 12 h	Significantly promote the production of iturin A, and the content of main antinutritional factors are greatly reduced	Jin et al. (2014)
		*Bacillus subtilis, Lactobacillus fermentum*	*Lactobacillus* fermentum and *Bacillus subtilis* were mixed at a ratio of 1:1 for fermentation	The content of isothiocyanate was significantly reduced and the nutrition was significantly improved	Xu et al. (2012)
		*Bacillus subtilis, Enterococcus faecium, Lactobacillus, Saccharomyces cerevisiae,*	Bacteria ratio of 1:1:1:1:1. rapeseed meal, wheat bran and 1% brown sugar was mixed for fermentation	Improve broiler performance, nutrient digestibility and rapeseed meal feeding amount, and maintain intestinal ecological health	Chiang et al. (2010)
		*Less spore rhizopus*	Solid fermentation	The contents of GS, OZT, PA and CF decreased by 43.1%, 34%, 42.4% and 25.5%, respectively	Vig et al. (2001)
		*Lactobacillus*	Adding wheat bran for fermentation	The solubility of protein, nitrogen, and phosphorus is significantly improved	Poulsen and Blaabjerg (2017)
		*Lactic Acid Bacteria (Pediococcus acidilactici, Pediococcus pentosaceus, Lactobacillus plantarum)*	Anaerobic solid fermentation	Improving the development of colonic mucosa and the maturity of intestinal flora of weaned piglets and maintain the intestinal health	Satessa et al. (2020)
		*White-rot fungi (Trametes versicolor, Pleurotus ostreatus*)	Solid fermentation	Both fungi can effectively decompose the antinutritional phenols in rapeseed meal	Tie et al. (2020)
		*Rhizopus oligosporus sp-*T3	Fermented for 40 h at 32 ℃, pH 5.0	84% of carbohydrates, 30% of lignin and other polyphenols and 47% of total GLS were degraded, significantly improved the nutritional value	Rozan et al. (1996)
Enzyme and microorganism	Acid proteinase	*Aspergillus niger*	*Aspergillus niger* solid-state fermentation (SSF). Then, After 48 h of fermentation at 30 ℃, enzymatic hydrolysis at 45 ℃ is 24 h	Can more effectively degrade the antinutritional factors	Tie et al. (2020)
	Laccase	*Basidiomycota fungus, Trametes* sp 48,424, *yeast Saccharomyces cerevisiae*	solid or liquid fermentation	It has obvious digestion effect on SE, and it is preliminarily	Niu et al. (2015)
	Lipase	*Bacillus amyloliquefaciens* CX-20	Adding 5% (v/v) exponential growth cells and lipase, 72 h	The amount of iturin A increased from 0.82 g/L to 1.14 g/L, which was 38.15% higher than that without lipase	Chen et al. (2019)

Niu et al. (2015) suggested that laccase is efficient at digesting SE in RSM. Tie et al. (2020) reported that the GLS degradation rate and changes in trichloroacetic acid-soluble protein content were associated with changes in endoglucanase activity. It is speculated that endoglucanase destroys the cellulose network structure of RSM, leading to the loss of protection and degradation of GLS and plant proteins embedded in its core. This suggests that endoglucanase plays an important role in improving RSM quality. Nibedita et al. (2007) found that the tannin removal rate from RSM was 61.25% under moisture content of 41.22%, temperature of 82.5 ℃, and extrusion speed of 90 rpm. *Bacillus subtilis* catalases CotA and Yjqc are resistant to H_2_O_2_, and function as synergistic catalysts for the degradation of sinapic acid and sinapine in RSM (Zhang et al. 2016a). Myrosinase activity peaked on the third day of rapeseed germination stage. The crude extract of 0.90 g myrosinase could be obtained from 1 g of *B. napus* sprouts by precipitation with 20–60% saturated ammonium sulfate. Treatment with 9.63 μg/g ascorbic acid and 26.68 μg/g EDTA resulted in a degradation rate of > 80% for GLS (Xie et al. 2022). The nitrilase BnNIT2, extracted from *B. napus,* can convert nitriles from GLS to carboxylic acid and NH_3_. Under the conditions of pH 5.0 and Fe^2+^, the degradation rate of nitriles from GLS reached ~ 80% (Zhang et al. 2022).

At present, the effect of enzyme combinations on detoxification and quality improvement of RSM appears to be superior to that of single enzymes. Moreover, enzymatic detoxification can be further enhanced using physical treatments, such as ultrasound, expansion, and extrusion (Nibedita et al. 2007; Jin et al. 2016). RMS samples were pretreated using four methods (extrusion, hot water, dilute acid, and dilute alkali) and three fungi (*pullulan brachyderm* Y-2311–1, *Fusarium venenatum* NRRL-26139, and *Trichoderma reesei* NRRL-3653). The optimal combination on cold-pressed RSM was pretreatment using extrusion and *Fusarium venenatum* NRRL-26139 fermentation. This method resulted in a protein content of 54.4%, and a decrease of neutral detergent fibre (NDF), GLS, and residual sugar content to 11.6%, 6.7 µmol/L/g, and 3.8%, respectively. This approach not only reduced GLS (up to 98%) and NDF (up to 65%) in RSM, but also increased protein content in RSM (up to 45%) (Croat et al. 2017). RSM contains 16–22% (wt/wt) pectin polysaccharides, cellulose, hemicellulose, and other non-starch polysaccharides (NPA). The effects of acid extrusion and commercial pectinase on NPA fermentation in RSM were compared. It was found that the addition of enzymes in raw RSM significantly increased the fermentation of NPA (38%) compared with acid treatment (Pustjens et al. 2014). Thus, the fermentability of RSM polysaccharides can be improved using different physical or chemical pretreatment methods combined with enzymatic hydrolysis. Pustjens et al. showed that weak acid pretreatment and commercial pectin hydrolase treatment resulted in the best digestion effect on RSM carbohydrates, yielding a total carbohydrate content of only 32% (Pustjens et al. 2012).

### Microbial fermentation

The microbial method refers to the use of naturally propagated microorganisms or artificially added microbial preparations that secrete related enzymes to effectively decompose antinutritional factors and other macromolecular substances through fermentation. Addition of just 10% of raw RSM in the diet can reduce the weight and egg quality of laying ducks; antinutritional factors in RSM are the main obstacle to increase the content of rapeseed as a feed additive (Tan et al. 2022). Ashayerizadeh et al. (2017) examined the effect of fermented soybean meal (FRSM) and RSM as feed additives for broilers. Compared with RSM, feeding FRSM significantly reduced the colonization of chicken organs by *Salmonella enterica* serovar Typhimurium and the heterophil/lymphocyte ratio, and significantly increased weight gain and the feed conversion rate of the broilers. Compared with lettuce meal, FRSM after solid-state fermentation by *Aspergillus niger* improved growth performance and nutrient digestibility in pigs and is a promising alternative protein in the pig industry (Shi et al. 2016). Red snapper was fed different contents of *A. oryzae*-fermented RSM instead of fish meal; it was found that at 25–50% rapeseed content, the replacement of fish meal by fermented rapeseed promoted the growth and utilization of nutrients, resulted in increased immune responses and antioxidant effects, and significantly enhanced the lysozyme, bactericidal, and peroxidase activities of red snapper (Dossou et al. 2018).

The microbial fermentation method mainly uses strains, such as *B. subtilis*, *Saccharomyces cerevisiae*, *A. niger*, and *Lactobacillus. B. subtilis*, *A. niger,* and yeasts are the most commonly used organisms (Table 2). Fermentation of RSM by these organisms, either singly or in combination, can significantly improve protein hydrolysis, resulting in higher peptide yields and better detoxification effects. Microbial fermentation of RSM is usually divided into solid and liquid fermentation. Generally, the effect of multi-bacterial fermentation is more prominent than that of single bacterium. In addition to bacterial liquid, microbial fermentation usually requires the addition of other substrates, such as KH_2_PO_4_. Generally, aerobic fermentation is performed first, followed by facultative anaerobic fermentation. In some studies, enzymes were added following bacterial fermentation to facilitate enzymolysis. Combined fermentation of RSM and wheat bran not only further improves the degree of protein hydrolysis in RSM, but also enhances the solubility of protein and phosphorus (Poulsen and Blaabjerg 2017; Xu et al. 2012). Some authors have used combined microbial fermentation with enzymatic hydrolysis to effectively remove antinutritional factors from RSM, leading to a further increase in nutritional value and palatability (Chen et al. 2019; Tie et al. 2020).

## Studies on high-value applications of RSM

The rich nutritional components of RSM have led to the development of many high-value products using the latest biotechnological developments. Further, these processes add value to the agricultural industrial wastes.

### Development of value-added products as fermentation raw materials

In 1990, Gattinger et al. (1990) reported that the yield of xylanase produced from RSM was similar to or better than that produced from other substrates. In 1994, Ebune et al. (1995) used RSM as raw material to produce 5000 U/kg of phytase via solid-state fermentation by *Aspergillus ficuum* NRRL 3135. Thereafter, Imandi et al. (2013) used RSM as a medium substrate to produce lipase by solid-state fermentation with the marine yeast *Yarrowia lipolytica* NCIM 3589, whereby lipase activity reached 57.89 U/gds after 4 days of fermentation. Freitas et al. (2013) used RSM as a culture medium to produce protease via solid-state fermentation with *A. oryzae*, where the derived protease activity was 5.8-fold higher than that under the initial conditions. The effects of supplementing culture medium with RSM on the production of important enzymes for biotechnology has been examined in cultures of the white-rot fungi *Cerrena unicolor*. In the presence of 3.5% wt/v RSM, the activities of chitinase, β-glucosidase, and laccase were increased by 4.1, 8.4, and 3.9 times, respectively. These results indicate that liquid deep fermentation of RSM is an inexpensive and effective method to produce chitinase, β-glucosidase, and laccase by *C. unicolor* (Jaszek et al. 2016)*.*

As a culture medium, RSM not only promotes enzyme preparation, but also produces other high-value biological products. In general, the high content of proteins, carbohydrates, and minerals in RSM cannot be absorbed by most microorganisms, such as industrial bacteria, yeasts, and microalgae. However, these nutrients can be made available to microorganisms by short-term solid fungal fermentation of RSM followed by enzymatic hydrolysis (Kiran et al. 2012). RSM hydrolysate was prepared by solid fermentation and fungal autolysis using *A. oryzae*, *Penicillium oxalate,* and *Neurospora crassa*. Alternative fermentation media with RSM hydrolysate and molasses were developed to produce omega-3 docosahexaenoic acid (DHA) at levels comparable to those of commercial media containing expensive glucose and yeast extracts. The total cost of DHA production can be greatly reduced by fermenting bacteria on this inexpensive and environment-friendly medium (Gong et al. 2015). Chen et al. (2011) used RSM as raw material to produce succinic acid using *Actinobacillus succinogenes*, in combination with pretreatment using dilute sulfuric acid and simultaneous pectinase saccharification. The best effect resulted in succinic acid concentrations of 23.4 g/L and 11.5 g/100 g dry matter, which translated to a productivity of 0.33 g/L/h. Yao et al. (2012) used RSM as raw material to synthesize 5.3 g/kg of iturin A and 51.3 g/kg of poly-c-glutamic acid, via solid-state fermentation with *B. subtilis*. Tadi et al. (2021) used RSM as culture medium to produce poly-(3-hydroxybutyrate) via fermentation with *Bacillus megaterium*, reducing production costs and improving the utilization rates of RSM. Solid-state fermentation based on rapeseed can yield probiotic-rich polymers, such as levan, that have the potential to replace antibiotics. These are novel compounds with a promising potential in the context of a growing functional food market and can promote animal health and the ban of antibiotics (Konkol et al. 2019).

### Rapeseed proteins as food sources

Rapeseed proteins are considered potential food additives that mainly exist in embryos as storage proteins, accounting for 80% of the total protein content. Napin (a 2 S albumin) and cruciferin (a 12 S globulin) are the two main protein storage families (Hoglund et al. 1992). Napin has good foaming performance and cruciferin acts as a gel agent. Oleosin content in rapeseed protein is 21.8%. It is a low-molecular weight (15–26 kDa) alkaline protein (Huang 1992). Rapeseed proteins are efficient in water absorption and retention, which can improve the water-binding ability of food and enhance flavour retention. The good emulsification (EC) property of rapeseed proteins is an important factor in their application in the food industry, with uses that include the manufacturing of milk and meat products, and stabilizing emulsions in salad dressings and mayonnaise. The EC value, foaming ability, and stability of RSM proteins were significantly higher than those of soybean meal and flaxseed meal; however, heat treatment denatured proteins and reduced these parameters (Khattab and Arntfield 2009). As a potential and promising source of bioactive compounds, RSM proteins can supply active peptides that inhibit angiotensin-I-converting enzyme (ACE). However, the content of this active peptide in treated RSM proteins is significantly lower than that in nontreated RSM proteins (Wu et al. 2009).

### Functional substances

#### Bioactive peptides in rapeseed proteins

Evidently, hydrolysis of rapeseed proteins produces peptides—with relatively small molecular weight—with biological activity. The components of these peptides are complex, and their biological activity is related to amino acid composition, structure, sequence, and molecular weight (Elias et al. 2008). The crude rapeseed peptides obtained by the digestion of rapeseed protein with Alcalase 2.4 L showed antithrombotic activity and a noticeable inhibitory effect on fibrinogen-induced coagulation catalysed by thrombin. When the concentration of peptide is 30–50 mg/mL, the inhibitory effect reaches 90% (Zhang et al. 2008). The antihypertensive peptide Arg-Ile-Tyr is obtained using the *B. subtilis* protease rapakinin, which can also inhibit the activity of ACE (at IC50 = 28 µM) (Yamada et al. 2010). The antitumour effects of rapeseed protein hydrolysates (RSCH), derived from RSM, were confirmed in vivo using the S180 tumour-bearing mouse model. The presence of RSCH may lead to improved immune function in mice by reducing the formation of free radicals and oxidative stress responses; importantly, death or growth retardation in mice were not observed when RSCH was administered at 150 mg/kg/d (Xue et al. 2009a, b). Wang et al. (2016) used solid-state fermentation of RSM to obtain the antitumour active peptide RSP-4-3-3, which significantly alters the morphological characteristics of HepG2d tumour cells in vitro, thereby inducing apoptosis and inhibiting their proliferation. Cobs-Rosas et al. (2015) found that pectin extracted from RSM showed antiproliferation activity against tumour cells; however, antiproliferative activity varied depending on pectin type and extraction process. The use of microorganisms to transform biomass and obtain derivatives of medicinal value is common. For example, Yu et al. (2017) obtained two artemisinin derivatives (1-deoxyartemisinin and alpha hydroxy 4-1-deoxyartemisinin) by the biotransformation of artemisinin using *Aspergillus terreus*.

#### Phenolic antioxidants

Rapeseed contains more phenolic compounds than any other oilseed plant, and most of the phenolic compounds remain in the RSM; as a result, the antioxidant activity of phenols is retained when RSM is used as feed. Several studies have confirmed the excellent antioxidant properties and great potential of phenolic compounds in the development of functional foods. The antioxidants in SA help prevent cardiovascular disease and inhibit histone deacetylase activity that has been associated with the development of diabetes (Senawong et al. 2013; Silambarasan et al. 2015, 2016; Cherng et al. 2013). Marta et al. (2018) reported that high concentrations of polyphenols extracted from RSM significantly reduced free advanced glycation end products. Elias et al. (2008) used *A. oryzae* and *Basidiomycetes squamae*, combined with green chemistry, to gently and effectively obtain high-value canolol from RSM. In addition, studies have verified that SA may play a role not only in the treatment of hypertension but also in the prevention and treatment of hypertension-related diseases, such as vascular hypertrophy, retinal diseases, and stroke (Harlan et al. 2015). Currently, existing synthetic ACE inhibitors, such as captopril and enalapril, have various side effects, including cough, rash, and vomiting. Therefore, SA from natural food sources has great potential for medical applications. In recent studies, SA has shown a potential synergistic effect with captopril. Such synergistic effect does not result from the inhibition of ACE but through endogenous vasodilators (Wang et al. 2020). At present, the strategies for extracting phenolic acids, including SA, often refer to the methods published by Naczk et al. (1992) methanol, propanol, and water (7: 7: 6) are used as solvents for free and esterified phenolic acid extraction. To improve the extraction of phenolic acids from RSM, accelerated solvent extraction and other technologies have also been used .

Phenolic substances in RSM have wide applications as antioxidants in the food industry, biodiesel production, and cosmetics. High-performance liquid chromatography methods (HPLD-DAD and HPLC–ESI–MS) have been used to identify the protein phenolic compounds of RSM that were converted to myrosinic acid (SA) by hydrolysis. Using DPPH and ABTS colorimetric tests revealed that SA had better antioxidant performance than sinapine(SP), even better than that of vitamin C (Le et al. 2012). These phenolic compounds in rapeseed have the potential to be used as natural antioxidants in the food industry. Vuorela et al. (2004) separated phenolic compounds from RSM using different methods and found that these compounds enhanced the scavenging of free radicals, inhibited the formation of liposomes and low-density lipoproteins, and showed superior antioxidant effects; thus, it is reasonable to consider them as new products in the food and cosmetic industries. Iguchi et al. (2021) found that high purity SE (100 wt%) could be obtained by preparative thin-layer chromatography and cation-exchange resin with adsorption and catalytic functions, using only ethanol and water as solvents; thus prepared, SE showed substantial antioxidant effect in oil preservation (Iguchi et al. 2021). The polyphenol mixture in RSM was extracted using 0.2% HClO_4_ in methanol/acetone (1: 1 v/v); the solvent extract could significantly inhibit the oxidation reaction and microbial growth of biodiesel, and delay the degradation and oxidation of biodiesel without affecting the main quality parameters (Manashi et al. 2013). Dry distillation is a promising alternative to solvent-assisted process and suitable for concentrating protein cellulose, lignin, and polyphenols from many agricultural resources. Compared with solid liquid fractionation, it has a higher energy efficiency and reduced environmental impact and can produce enrichment fractions with natural function. Oscar et al. (2018). recovered high protein and phenolic fractions from RSM and sunflower meal using two separate techniques based on particle charge (electrostatic separation) and density (turbine separation), with an overall recovery of 30%.

### Production of bioenergy

In recent years, increased attention to the safety of energy supplies, climate change, and environmental protection, has stimulated interest in the use of biomass to produce bioenergy (Luo et al. 2011). Kiran et al. (2013) found that the solid-state fermentation of RSM by *A. oryzae* and autolysis by filamentous fungi can be used as a low-cost method for the production of microbial bio-oil by the *Rhodosporidium toruloides* yeast. This method offered a better carbon and nitrogen balance for lipid accumulation, and the derived, highly unsaturated lipids could be used for biodiesel production. Production of polyhydroxyalkanoate (PHA) based on RSM hydrolysates may replace expensive carbon sources, nutritional supplements, and precursors for copolymer production, integrating the bioconversion and production of PHA into existing bio-oil production. The method has the potential to enhance the feasibility and sustainability of the first-generation biorefineries. García et al. (2013) used RSM hydrolysate to supplement PHA production, although glycerol was the main substrate. Wongsirichot et al. (2020) used RSM hydrolysates as the PHA production medium for *Pseudomonas putida*. The authors showed that nitrogen-rich RSM and short-term oxygen supply could effectively induce PHA accumulation. Qian et al. (2013), using methanol and NaOH to perform in situ alkaline transesterification reaction on RSM, managed to produce biodiesel at a 98% conversion rate and, at the same time, reduce the content of GLS in the remaining RSM to 0.07%. Thus, considering biodiesel production and RSM detoxification, the overall cost of biodiesel production can be reduced and the problem of protein feed shortage can be alleviated (Qian et al. 2013).

### Environmental biological repair and protection

RSM-based applications are diversifying, not only in the fermentation sector but also in energy production, medical, and other fields. Moreover, RSM shows potential for applications in biological repair, environmental protection, food, cosmetics, and the production and development of new materials. Zhou et al. (2015) showed that soil pH, soluble organic carbon, and organic nitrogen can be improved by planting *Sedum plumbizincicola* in polluted acidic soils, followed by the planting of rice and the application of RSM. This practice substantially reduced the content of heavy metals in brown rice crops and increased yields, thereby providing a new strategy to ensure safety in food production and bioremediation. Mazurek et al. (2021) pyrolysed RSM under anoxia and 973.15 K for 2 h to produce biochar, the specific surface area (166.99 m^2^/g) of which was superior to most reported biochar. Biochar can successfully separate Cu (II) and Zn (II) in industrial wastewater and shows excellent adsorption capacity (52.2 mg/g) for Cu (II) in a short equilibrium time (Mazurek et al. 2021). Biofumigation has attracted increasing interest as a method of controlling agricultural pests. In biofumigation, glucosinolate-rich plants are used as cover crops in the field; these release toxic secondary glucosinolate-rich byproducts that can lead to the reduction of pest, disease, and weed occurrence in cultivated and horticultural crops (Ngala et al. 2015; Bellostas et al. 2007). Regarding the extraction of GLS, cold methanol extraction is as effective or better than other methods in extracting GLS: it does not require the use of freeze dryers or boiling methanol, and thus it is less harmful and costly (Doheny-Adams et al. 2017).

### Other applications

Damian et al. found that the bioconversion of RSM using bacteria and yeast produces polymers and biosurfactants with high added value. These polymers can lead to the broad use of probiotics as substitutes for antibiotics in animal feed and are suitable for use in cosmetics due to excellent moisturizing properties. Biosurfactants display strong antibacterial effects and can be used to preserve feed (Konkol et al. 2019). Zhu and Wu (2009) extracted two main polysaccharide components from RSM: WPS-1 and APS-2. Polysaccharides, which are mainly formed from the polymerization of galactose, arabinose, and glucose, exert a strong scavenging effect on superoxide and hydroxyl radicals (Zhu and Wu 2009). Rivera et al. (2015) hydrolysed RSM using protease to obtain short-chain bioactive peptides that displayed good antioxidant, anti- wrinkle, and anti-inflammatory activities; the peptides were safe, nontoxic, and compatible with skin fibroblasts, thereby showing good application potential in skin care. Paciorek-Sadowska et al. (2019) used rapeseed cake as a biofiller in the production of rigid polyurethane–polyisocyanurate foams, which are considered the best polymer materials for heat insulation. Although the apparent density of foam material treated with rapeseed-cake grinds is higher than that without the biomaterial foam, its water absorption and compressive properties are improved, and it is less brittle and flammable (Paciorek-Sadowska et al. 2019). Rapeseed protein can also be used as a source of a new type of polymer membrane that exhibits thermoplasticity due to vitrification in the presence of plasticizers, such as glycerol, polyethylene glycol, and sorbitol. Rapeseed proteins have comparable mechanical properties and moisture resistance to other plant protein-based bioplastics and great potential in food packaging applications (Zhang et al. 2016b).

## Conclusions and future prospects

Currently, a satisfactory detoxification process that is suitable for use at the level of industrial production is lacking. The available data indicate that future research must focus on the effects of enzyme addition and microbial fermentation on the removal of antinutritional factors. Enzyme systems and microbial fermentation have shown outstanding effects on the removal of antinutritional factors and are considered potential strategies for RSM commercial feed production. With the development of rapeseed genetics, breeding, and biotechnology, it is expected that the antinutritional factors of RSM will be reduced more effectively; however, this route is relatively long. Domestication and culture of relevant strains as well as screening and development of more effective enzyme systems should be given priority. In parallel, generally recognized as safe (GRAS) bacteria and enzymes for rapeseed fermentation do not always yield the desired results, and the production costs and unresolved safety issues still need to be addressed.

The high-value applications of RSM is another important aspect of RSM resource development. Through biotransformation and advanced extraction technology, the added value of agro-industrial waste can be effectively increased, and products of high added value can be developed, providing new pathways for the application of RSM. Many published reports have highlighted the value of these substances, but there is no relatively mature technology for the effective extraction and preparation of active substances yet. Therefore, it is necessary to further study and identify effective methods for the extraction and preparation of materials that are conducive to promoting the high application value of active ingredients in the industry. It is expected to promote the high-value industrial application of RSM active ingredients by effective and advanced technology research aimed at the extraction and preparation of highly efficient active substances.

In the future, rapeseed products with high nutritional value and low toxicity are expected to play a greater role and make important contributions to alleviate the lack of protein resources in the global feed industry. Meanwhile, further development of technologies associated with RSM-based applications will enable RSM resources to be fully utilized and valued in many fields.

## Data Availability

Not applicable.

## Abbreviations

ACE: Angiotensin-I-converting enzyme
CpHe: Cold-pressed hexane extraction
DHA: Docosahexaenoic acid
EC: Emulsification
FRSM: Fermented soybean meal
GLS: Glucosinolates
GRAS: Generally recognized as safe
HPLD: High-performance liquid chromatography
ITC: Isothiocyanate
NDF: Neutral detergent fibre
NPA: Non-starch polysaccharides
OZT: Oxazolidine thione
PHA: Polyhydroxyalkanoate
RCN: Nitriles
RSCH: Rapeseed protein hydrolysates
RSM: Rapeseed meal
ScCO2: Supercritical carbon dioxide extraction
SE: Sinapine
TC: Thiocyanate
